# Electrolysis of water is an effective source of oxygen at high altitude

**DOI:** 10.1186/s12967-022-03443-2

**Published:** 2022-05-26

**Authors:** Xinjun Tang, Li Liang, Lijuan Hu, Yuanlin Song

**Affiliations:** 1 Department of Pulmonary and Critical Care Medicine, Zhongshan Hospital, Fudan University, Shanghai, China; 2 Shanghai Respiratory Research Institute, Shanghai, China; 3 Shanghai Key Laboratory of Lung Inflammation and Injury, Shanghai, China; 4 Department of Oncology, Zhongshan Hospital, Fudan University, Shanghai, China

## To the editor:

With the increasing number of unacclimatized lowlanders traveling to high altitude, the importance of the management of acute high altitude illness is growing. Acute high altitude illness is triggered by hypobaric hypoxia and can be fatal if not treated promptly [1]. Oxygen supplementation is lifesaving, but this is often impossible in resource-limited regions. Hydrogen/oxygen mixed gas, generated by electrolysis of water, has been proved safe and beneficial for various medical purposes [2, 3]. Compared with conventional oxygen supplementation, generating oxygen by electrolysis of water is cost-effective, environment-friendly, and easily obtainable, and we hypothesize that it would be a promising approach to provide oxygen at high altitude.

**Fig. 1:**
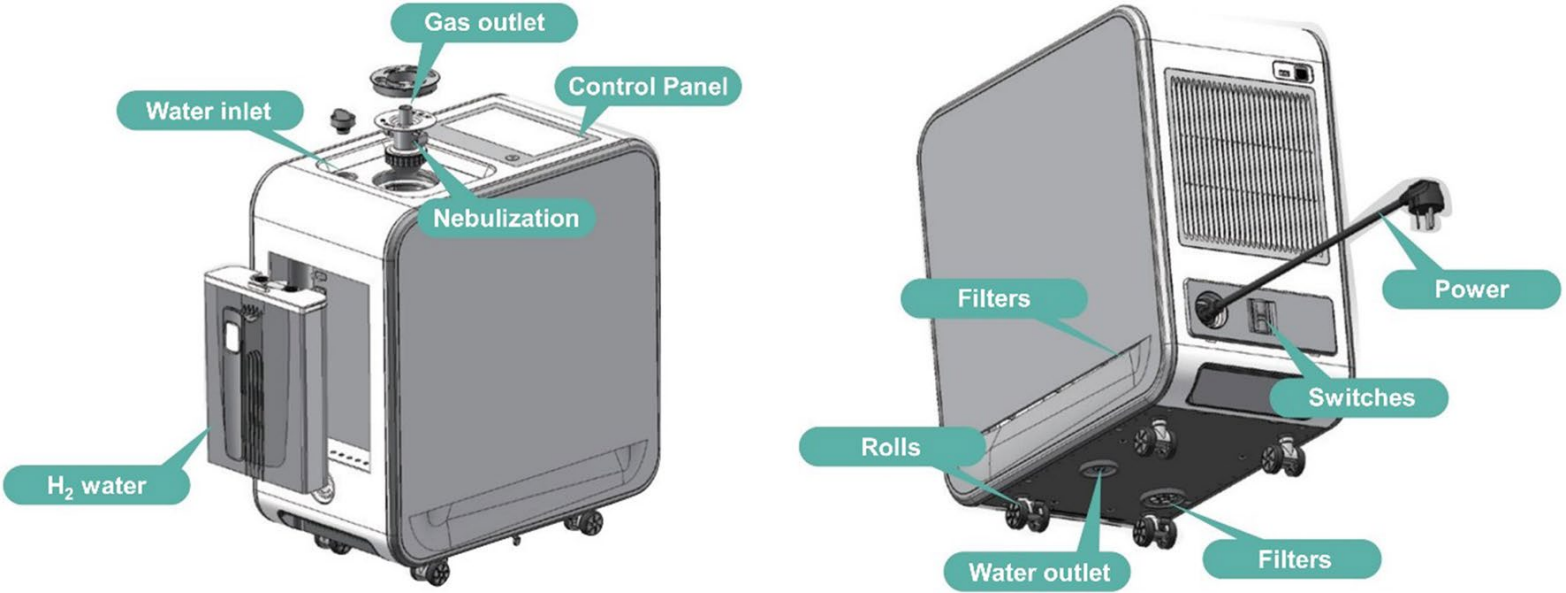
A schematic diagram of the Hydrogen/Oxygen Generator (model AMS-H-03). Courtesy of Shanghai Asclepius Meditec Co., Ltd. (Shanghai, China)

From May 1, 2021 to Oct 31, 2021, a male, 37-year-old physician from Shanghai (named as ‘the participant’ thereafter) was sent to work in Tibet for medical assistance. Before departure, he underwent a comprehensive medical examination to ensure his work competence at high altitude. After a period of acclimatization, the participant stayed at Gamba County (about 4700 m above sea level) on working days, and stayed at Rikaze City (about 3800 m above sea level) on rest days. When the participant was in Gamba County, he inhaled hydrogen/oxygen mixed gas (66% hydrogen; 33% oxygen) at 3 L/min via nasal cannula by using the Hydrogen/Oxygen Generator (AMS-H-03, Shanghai Asclepius Meditec Co., Ltd., China) (Fig. 1) for 15 h daily (from about 6 p.m. to 9 a.m. the next day). While resting in Rikaze City, he received conventional oxygen supplementation at 2 L/min via nasal cannula for 15 h daily (from about 6 p.m. to 9 a.m. the next day). Peripheral saturation of oxygen (SpO_2_), pulse rate, and apnea–hypopnea index (AHI) were detected by wearable polysomnography (SF-A8, VentMed Technology, China).

Data collection began from Jul 1, 2021. As of Oct 31, 2021, data of 34 days of inhaling hydrogen/oxygen mixed gas and 51 days of conventional oxygen supplementation was collected. The difference of SpO_2_ between inhaling hydrogen/oxygen mixed gas and conventional oxygen supplementation was almost statistically significant (P = 0.050), yet the gap (93.2% vs. 94.0%) was small. There was no significant difference in pulse rate (61.0/min vs. 61.2/min, P = 0.335) and AHI (13.7 vs. 12.6, P = 0.280) (Table 1).

**Table 1:** Polysomnography of the participant

	Hydrogen/oxygen mixed gas (34 days)	Conventional oxygen supplementation (51 days)	P*
Location	Gamba County	Rikaze City	
Altitude	About 4700 m	About 3800 m	
SpO_2_ (%)	93.2 [91.5, 94.2]	94.0 [92.7, 94.6]	0.050
Pulse rate (/min)	61.0 [59.7, 62.3]	61.2 [59.5, 63.2]	0.335
AHI	13.7 [10.0, 18.9]	12.6 [8.8, 17.2]	0.280

SpO_2_: peripheral saturation of oxygen; AHI: apnea-hypopnea index

*Compared by Mann–Whitney U test

Our study firstly demonstrated that hydrogen/oxygen mixed gas is an effective source of oxygen at high altitude. In our study, there was no significant difference in pulse rate and AHI between the two groups. In this context, the medium SpO_2_ was 93.2% when inhaling hydrogen/oxygen mixed gas, and was 94.0% when receiving conventional oxygen supplementation. Given that the participant was at a higher altitude when inhaling hydrogen/oxygen mixed gas, we assume that the effect of oxygen therapy of both method would be similar at same altitude, although the actual oxygen supply of the Hydrogen/Oxygen Generator was half of conventional oxygen supplementation. One possible explanation is that gaseous hydrogen has a much smaller density than the air, hence a much lower airway resistance when passing through the respiratory tract [3]. A new technology, oxygen conditioning, has been proved practicable in raising the oxygen concentration of the air and, therefore, the living and working environment at high altitude [4]. For every 1% increase in the oxygen concentration, the physiological altitude is reduced by approximately 300 m [5]. The commonest way of generating oxygen for medical purpose is to use synthetic zeolites, which collect oxygen-enriched gas by absorbing nitrogen under appropriate pressure conditions. However, this is expensive and requires large amount of synthetic zeolites. Since water is cheap and easily obtainable, electrolysis of water is cost-effective to generate enough oxygen for medical purpose.

Before the clinical use of hydrogen, helium has been proved effective in the management of obstructive lung disease for its low density. However, its expensiveness limits its widespread use. Gaseous hydrogen and helium have the same molecular weight, making it possible in clinical settings. As hydrogen is combustible, people may question about its safety and tolerability, especially in an oxygen-enriched environment. In our study, no adverse event was observed, similar to previous studies at normal barometric pressure [2, 3]. A previous study has tested the safety of the Hydrogen/Oxygen Generator, and revealed that the maximum hydrogen concentration was far below the explosion limits of hydrogen in the air (4%) [3]. Furthermore, oxygen conditioning at high altitude never raises the partial pressure of oxygen in the air above the normal sea level value. As a consequence, oxygen conditioning can reduce the physiological altitude without increasing a fire hazard [4].

In summary, we demonstrated that hydrogen/oxygen mixed gas inhalation is effective in oxygen supplementation at high altitude. Since water is low-cost and easily accessible, electrolysis of water by the Hydrogen/Oxygen Generator is promising for the treatment and prevention of high altitude illness. In addition, electrolysis of water has the potential to become a breakthrough in oxygen conditioning to change the living and working conditions at high altitude.

## Data Availability

The datasets used and/or analyzed during the current study are available from the corresponding author on reasonable request.
